# Wireless Sensor Networks Intrusion Detection Based on SMOTE and the Random Forest Algorithm

**DOI:** 10.3390/s19010203

**Published:** 2019-01-08

**Authors:** Xiaopeng Tan, Shaojing Su, Zhiping Huang, Xiaojun Guo, Zhen Zuo, Xiaoyong Sun, Longqing Li

**Affiliations:** College of Artificial Intelligence, National University of Defense Technology, Changsha 410073, China; tanxiaopeng14@nudt.edu.cn (X.T.); susj-5@163.com (S.S.); kdhuangzp@163.com (Z.H.); z.zuo@nudt.edu.cn (Z.Z.); sxy15084973192@163.com (X.S.); 15575982374@163.com (L.L.)

**Keywords:** wireless sensor networks, intrusion detection, class imbalance, SMOTE, random forest

## Abstract

With the wide application of wireless sensor networks in military and environmental monitoring, security issues have become increasingly prominent. Data exchanged over wireless sensor networks is vulnerable to malicious attacks due to the lack of physical defense equipment. Therefore, corresponding schemes of intrusion detection are urgently needed to defend against such attacks. Considering the serious class imbalance of the intrusion dataset, this paper proposes a method of using the synthetic minority oversampling technique (SMOTE) to balance the dataset and then uses the random forest algorithm to train the classifier for intrusion detection. The simulations are conducted on a benchmark intrusion dataset, and the accuracy of the random forest algorithm has reached 92.39%, which is higher than other comparison algorithms. After oversampling the minority samples, the accuracy of the random forest combined with the SMOTE has increased to 92.57%. This shows that the proposed algorithm provides an effective solution to solve the problem of class imbalance and improves the performance of intrusion detection.

## 1. Introduction

The wireless sensor network is a distributed intelligent network system. It is composed of a large number of micro sensor nodes deployed in the detection area, which have the ability of wireless communication and computing. It can accomplish the assigned tasks independently according to the changes of environment. With the rapid development of wireless sensor technology, embedded computing technology, wireless communication technology, and distributed information processing technology, wireless sensor networks have very broad application prospects, such as national defense, ecological observation, environmental monitoring, medical security, space exploration, volcano observation, architecture, and city management, etc. [1,2,3,4].

Wireless sensor networks can realize real-time monitoring, sensing and collecting information of various environments or monitoring objects through the cooperation of various integrated micro-sensors. Then the information is processed by embedded system. Finally, the perceived information is transmitted to the user terminal by multi-hop relay through random self-organizing wireless communication network. In this process, the sensor nodes are located in a large area without protection or in a harsh environment, which makes them easy to be captured and leak sensitive information. Excessive security mechanisms of wireless sensor networks is not suitable for resource-constrained sensor networks. The characteristics of wireless jump communication make it easier to be eavesdropped, jammed, and attacked. The low cost of sensor nodes also makes it easy for the nodes to leak the key after being captured, which will lead to the insecurity of the whole network [5,6,7,8].

Currently, the security of wireless sensor networks has become a major concern. How to identify various network attacks is a key technology that needs to be solved [9,10]. The security studies of wireless sensor networks mainly focuses on the following aspects: (1) various network attack models and defense strategies; (2) encryption algorithms, key management, and authentication technology; (3) network routing and the security of data fusion; and (4) network intrusion detection systems and response models [11,12]. The security studies of wireless sensor networks can be divided into passive defense and active defense. Compared with the advancement of passive defense studies for wireless sensor networks, there are too few studies on active defense. However, passive defense is the measure taken in response to the characteristics of the attacks after the attacks have occurred, and this is not sufficient for the security of wireless sensor networks. Therefore, it is urgent to study active defense technologies to detect malicious intrusions before attacks occur. As an active defense technology, intrusion detection will play an important role in ensuring the security of wireless sensor networks [13,14,15,16,17]. This paper will focus on introducing intrusion detection technology in wireless sensor networks.

In the past few years, many methods have been proposed to design intrusion detection systems for wireless sensor networks. Lu et al. [18] introduced an evolutionary mechanism to extract intrusion detection rules. In order to extract diverse rules and control the number of rule sets, rules are checked and extracted according to the distance between rules in the same type of rule set and rules in different types of rule sets. Singh et al. [19] proposed an advanced hybrid intrusion detection system (AHIDS) that automatically detects wireless sensor networks attacks. AHIDS utilizes the cluster architecture and enhanced LEACH protocol to reduce the energy consumption of sensor nodes. AHIDS uses anomaly detection and misuse detection based on a fuzzy rule set and a multi-layer perceptron neural network. Due to the advantages of the negative selection algorithm (NSA) in the classification domain, Sun et al. [20] proposed a WSN-NSA intrusion detection model based on the improved V-detector algorithm for wireless sensor networks (WSN). The V-detector algorithm is modified by modifying detector generation rules and optimizing detectors, and principal component analysis is used to reduce detection features. Tajbakhsh et al. [21] proposed an intrusion detection model based on fuzzy association rules, which uses fuzzy association rules to construct classifiers, and uses some matching metrics to evaluate the compatibility of any new samples with different rule sets. And the class corresponding to the best matching rule set is declared as the label of the sample. Xie et al. [22] focus on detecting a special type of anomaly in wireless sensor network (WSN), which appears simultaneously in a collection of neighboring nodes and lasts for a significant period of time. With the proposed distributed segment-based recursive kernel density estimation, a global probability density function can be tracked and its difference between every two periods of time is continuously measured for decision making. Xie et al. [23] focus on a new technique for handling data in a segment-based manner. Considering a collection of neighboring data segments as random variables, they determine those behaving abnormally by exploiting their spatial predictabilities and, motivated by spatial analysis, specifically investigate how to implement a prediction variance detector in a WSN. Haider et al. [24] proposed a metric using a fuzzy logic system based on the Sugeno fuzzy inference model for evaluating the quality of the realism of existing intrusion detection system datasets. Based on the proposed metric results, they designed and generated a synthetically realistic next-generation intrusion detection system dataset, and a preliminary analysis was conducted to assist in the design of future intrusion detection systems.

At present, many intrusion detection systems for wireless sensor networks are rule-based systems whose performance is highly dependent on the rules determined by security experts. Due to the vast amount of network traffic, the process of encoding rules is both expensive and slow. In order to overcome the limitation of rule-based system, data mining technology is used in intrusion detection systems for wireless sensor networks. Data mining is a successful solution for active detection of network attacks based on features hidden in the data of network behavior. Through the analysis of large datasets, understandable patterns or models are explored, the intrusion patterns for anomaly detection are effectively extracted, and the classifiers used to detect attacks are constructed [25,26]. The intrusion detection system based on data mining is more flexible and easier to deploy. In this paper, an intrusion detection model for wireless sensor networks is proposed. A data mining algorithm called random forest is applied in intrusion detection for wireless sensor networks. The random forest algorithm is an ensemble classification and regression method, and it is one of the most effective data mining technologies [27,28].

One of the challenges of intrusion detection for wireless sensor networks is the imbalance of intrusion, such as Denial of Service (DoS) attacks, which have more connections than probing attacks, user to root (U2R) attacks, and root to local (R2L) attacks. Most data mining algorithms attempt to minimize the overall error rate, but this will increase the error rate for identifying minority intrusions. In the actual wireless sensor network environment, minority attacks are more dangerous than the majority of attacks. Considering the serious class imbalance of the intrusion dataset, synthetic minority oversampling technology (SMOTE) is adopted to balance the dataset, which effectively improves the detection performance of minority intrusions.

Lee et al. [29] proposed a hybrid approach for real-time network intrusion detection systems (NIDS). They adopt the random forest (RF) for feature selection. RF provides the variable importance by numeric values so that the irrelevant features can be eliminated. The experimental results show the proposed approach is faster and more lightweight than the previous approaches while guaranteeing high detection rates so that it is suitable for real-time NIDS. Singh et al. [30] used the parallel processing power of Mahout to build random forest-based decision tree model, which was applied to the problem of peer-to-peer botnet detection in quasi-real-time. The random forest algorithm was chosen because the problem of botnet detection has the requirements of high accuracy of prediction, ability to handle diverse bots, ability to handle data characterized by a very large number and diverse types of descriptors, ease of training, and computational efficiency. Ronao et al. [31] proposed a random forest with weighted voting (WRF) and principal components analysis (PCA) as a feature selection technique for the task of detecting database access anomalies. RF exploits the inherent tree-structure syntax of SQL queries, and its weighted voting scheme further minimizes false alarms. Experiments showed that WRF achieved the best performance, even on very skewed data.

Taft et al. [32] applied SMOTE as an enhanced sampling method in a sparse dataset to generate prediction models to identify adverse drug events (ADE) in women admitted for labor and delivery based on patient risk factors and comorbidities. By creating synthetic cases with the SMOTE algorithm and using a 10-fold cross-validation technique, they demonstrated improved performance of the naïve Bayes and the decision tree algorithms. Sun et al. [33] proposed a new imbalance-oriented SVM method that combines the synthetic minority over-sampling technique (SMOTE) with the Bagging ensemble learning algorithm and uses SVM as the base classifier. It is named as the SMOTE-Bagging-based SVM-ensemble (SB-SVM-ensemble), which is theoretically more effective for financial distress prediction (FDP) modeling based on imbalanced datasets with a limited number of samples. Santos et al. [34] proposed a new cluster-based oversampling approach robust to small and imbalanced datasets, which accounts for the heterogeneity of patients with hepatocellular carcinoma, and a representative dataset was built based on K-means clustering and the SMOTE algorithm, which was used as a training example for different machine learning procedures.

The intrusion detection for wireless sensor networks mainly solves the classification problem of normal data and attack data. To improve the situation of class imbalance of the dataset, this paper proposed a classification method of random forest combined with the SMOTE. A random forest is a combined classifier that uses the method of resampling to extract multiple samples from the original samples and trains the sample set obtained by each sampling to establish a decision tree. Then these decision trees is combined together to form the random forest. The classification results of each decision tree are combined by voting to finally complete the classification prediction. The main content of the SMOTE is to insert new samples that are generated randomly between minority class samples and their neighbors, which can increase the number of minority class samples. In this paper, SMOTE is used to oversample the dataset, then the training set is reconstructed and the original data of class imbalance is balanced. After that, the random forest algorithm is used to train the new training set and the classifier is generated to realize the intrusion detection for wireless sensor networks.

The remaining sections of this paper are organized as follows. Section 2 describes the principle of SMOTE. Section 3 describes the random forest algorithm. Section 4 describes the intrusion detection technology combined with SMOTE and random forest. Section 5 describes experimental results and analysis, including dataset, evaluation indicators, results and comparison. Finally, Section 6 summarizes the paper.

## 2. Principle of SMOTE

Synthetic minority oversampling technology (SMOTE) is a heuristic oversampling technique proposed by Chawla et al. to solve the problem of class imbalance [35]. It has significantly improved the situation of over-fitting caused by non-heuristic random oversampling method, so it has been widely used in the field of class imbalance in recent years. The core idea of SMOTE is to insert new samples that generated randomly between minority class samples and their neighbors, which can increase the number of minority class samples and improve the situation of class imbalance [36,37,38,39].

Firstly, the *K* nearest neighbors are searched for each data sample *X* in the minority class samples. Assuming that the sampling magnification of the dataset is *N*, *N* samples are randomly selected from K nearest neighbors (there must be *K* > *N*), and the *N* samples are recorded as $y_{1},y_{2},\cdots ,y_{N}$. The data samples *X* and $y_{i}$ are correlated, and the corresponding random interpolation operation is performed by the correlation formula between *X* and $y_{i}(i=1,2,\cdots ,N)$ to obtain the interpolated sample $p_{i}$, so that *N* corresponding minority class samples are constructed for each data sample.

The interpolation formula is as follows:

(1) $$p_{i}=X+rand(0,1)\times (y_{i}-X),i=1,2,\cdots ,N$$

where *X* represents a data sample in minority class samples, rand(0,1) represents a random number within the interval (0,1), and $y_{i}$ represents the ith of the *N* nearest neighbors of the data sample *X*.

The sampling magnification *N* depends on the imbalanced degree of the dataset. The formula for calculating the imbalanced level (*IL*) between the majority class and the minority class of the dataset is as follows:

(2) N=round(IL)

where round(IL) represents the value obtained by rounding up the *IL*. Through the above interpolation operation, the majority class samples and the minority class samples can be effectively balanced, thereby improving the classification accuracy of imbalanced datasets.

**Figure 1:**
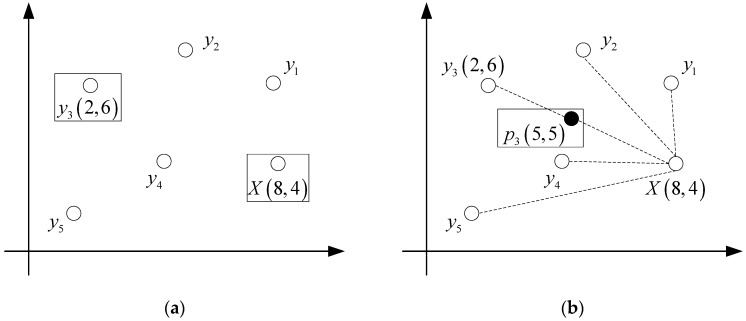
(**a**) The data sample *X* and its five nearest neighbors; and (**b**) interpolation principle of SMOTE.

In order to represent the interpolation process of SMOTE, it is assumed that there is a two-dimensional dataset, taking one of the data sample points *X*, whose coordinates are (8,4). The random value of rand (0,1) is set to 0.5, and the coordinates of a nearest sample point of *X* are set to (2,6). The representations of data sample *X* and its five nearest neighbors is shown in Figure 1a.

Figure 1a shows that the data sample *X*(8,4) has obtained its five nearest neighbors $\left(y_{1},y_{2},y_{3},y_{4},y_{5}\right)$, and now the sampling operation between *X* and the neighbor $y_{3}$ is performed.

According to Equations (1) and (2), the following results can be obtained:

(3) $$p_{3}=X+rand(0,1)\times (y_{3}-X)=(8,4)+0.5\times ((2,6)-(8,4))=(5,5)$$

That is, the newly generated interpolation is $p_{3}(5,5)$.

The whole interpolation process for generating new data is represented on the two-dimensional axis as shown in Figure 1b. It can be seen from the figure that the sampling of SMOTE is a random interpolation operation on the line between the data sample and its nearest neighbor. This method can be regarded as a linear interpolation, but its effect has been greatly improved compared with the simple replication of original data samples.

**Figure 2:**
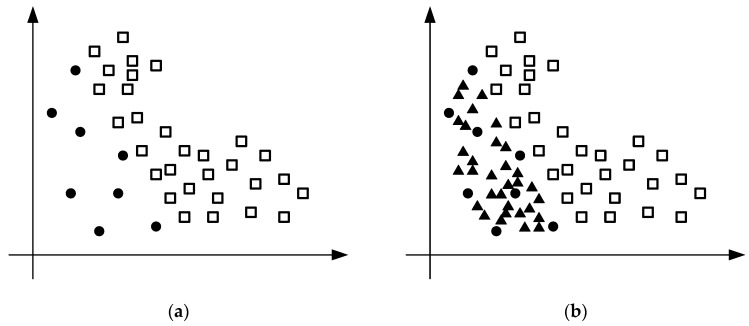
(**a**) Imbalanced dataset; and (**b**) entirely oversampling.

Now consider a more obvious imbalanced dataset. Suppose that there are 30 samples in the majority class and eight samples in the minority class. The distribution of the dataset is shown in Figure 2a. It can be seen from the figure that the difference between the majority class samples and the minority class samples is large. If data classification is performed in this case, the accuracy of data classification will be seriously reduced. Therefore, SMOTE is used to oversample the imbalanced data. According to the principle of SMOTE and the Equation (1), it can be known that when the sampling magnification is 4, it is possible to make the minority class samples reach the same number as the majority class samples.

The effect of oversampling the whole imbalanced dataset with SMOTE is shown in Figure 2b. The circle represents minority class, the square represents majority class, and the triangle represents synthetic data. It can be seen from the figure that for each minority class sample, four minority class samples of its nearest neighbors are selected for interpolation operation, and all interpolations are on a certain line between the original minority class sample and its nearest neighbor.

Through the analysis of SMOTE and the analysis of the imbalanced dataset before oversampling, it can be seen that the oversampling algorithm based on SMOTE has the following advantages. Firstly, SMOTE reduces the limitations and blindness of the oversampling algorithm for imbalanced data in the sampling process. The sampling method before SMOTE is a random upward sampling method, which can balance the dataset, but the sampling effect is not ideal because of the serious lack of principle of random sampling. The basic mathematical theory of linear interpolation is adopted by SMOTE. For data sample *X*, *K* samples of its nearest neighbors are selected, and then data are generated purposefully according to certain mathematical rules, which can effectively avoid blindness and limitations. Secondly, SMOTE effectively reduces the phenomenon of over-fitting. The method of replicating data is adopted by the traditional over-sampling technology. Since the decision domain becomes smaller in the sampling process, it leads to over-fitting. SMOTE can effectively avoid this defect.

## 3. Random Forest Algorithm

Random forest is an ensemble learning model which takes decision tree as a basic classifier. It contains several decision trees trained by the method of Bagging [40]. When a sample to be classified is entered, the final classification result is determined by the vote of the output of a single decision tree. Random forest overcomes the over-fitting problem of decision trees, has good tolerance to noise and anomaly values, and has good scalability and parallelism to the problem of high-dimensional data classification. In addition, random forest is a non-parametric classification method and driven by data. It trains classification rules by learning given samples, and does not require prior knowledge of classification.

The random forest model is based on *K* decision trees. Each tree votes on which class a given independent variable *X* belongs to, and only one vote is given to the class it considers most appropriate [41,42,43]. The description of the *K* decision trees is as follows:

(4) $$\left\{h(X,\theta_{k}),k=1,2,\cdots ,K\right\}$$

Among them, *K* is the number of decision trees contained in random forests. $\theta_{k}$ represents independent and identically distributed random vectors.

The steps to generate a random forest are as follows:

1. The method of random repeated sampling is applied to randomly extract *K* samples from the original training set as self- service sample set, and then *K* classification regression trees are generated.
2. Assuming that the original training set has *n* features, *m* features are randomly selected at each node of each tree (m≤n). By calculating the amount of information contained in each feature, a feature with the most classification ability is selected among the *m* features for node splitting.
3. Every tree grows to its maximum without any cutting.
4. The generated trees are composed of random forest, and the new data is classified by random forest. The classification results are determined by the number of votes of the tree classifiers.

The similarity and correlation of decision trees are important features of random forest to reflect generalization performance, while generalization error reflects generalization ability of the system. Generalization ability is the ability of the system to make correct judgments on new data with the same distribution outside the training sample set. Smaller generalization error can make the system get better performance and stronger generalization ability.

The generalization error is defined as follows:

(5) $$PE^{*}=P_{X,Y}(mr(X,Y)<0)$$

where $PE^{*}$ represents the generalization error, the subscript *X*, *Y* indicates the definition space of the probability, and mr(X,Y) is the margin function.

The margin function is defined as follows:

(6) $$mr(X,Y)=avg_{k}I(h(X,\theta_{k})=Y)-\max_{J\neq Y}avg_{k}I(h(X,\theta_{k})=J)$$

where *X* is the input sample, *Y* is the correct classification, and *J* is the incorrect classification. I(g) is an indicative function, $avg_{k}(g)$ means averaging, and h(g) represents a sequence of classification model. The margin function reflects the extent to which the numbers of votes for the correct classification corresponding to sample *X* exceeds the maximum number of votes for other incorrect classifications. The larger the value of margin function is, the higher the confidence of the classifier will be.

The convergence expression of generalization error is defined as follows:

(7) $$\lim_{k\to \infty }PE^{*}=P_{X,Y}(P_{\theta }(I(h(X,\theta_{k})=Y))-\max_{J\neq Y}P_{\theta }(I(h(X,\theta_{k})=J)))$$

The above formula indicates that the generalization error will tend to an upper bound, and the model will not over-fit with the increase of the number of decision trees.

The upper bound of the generalization error is available, depending on the classification strength of the single tree and the correlation between the trees. The random forest model aims to establish a random forest with low correlation and high classification intensity. Classification intensity *S* is the mathematical expectation of mr(X,Y) in the whole sample space:

(8) $$S=E_{X,Y}mr(X,Y)$$

θ and $\theta^{\prime }$ are independent and identically distributed vectors, and the correlation coefficients of mr(θ,X,Y) and $mr(\theta^{\prime },X,Y)$ is defined as follows:

(9) $$\bar{\rho }=\frac{{\mathrm{cov}}_{X,Y}(mr(\theta ,X,Y),mr(\theta^{\prime },X,Y))}{sd(\theta )sd(\theta^{\prime })}$$

Among them, sd(θ) can be expressed as follows:

(10) $$sd(\theta )=\sqrt{\frac{1}{N}\sum_{i=1}^{N}{\left(mr\left(x_{i},\theta \right)-\frac{1}{N}\sum_{i=1}^{N}mr\left(x_{i},\theta \right)\right)}^{2}}$$

In Equation (9), the correlation between the trees of h(X,θ) and $h(X,\theta^{\prime })$ on the dataset of *X* and *Y* can be measured by the $\bar{\rho }$. The larger the $\bar{\rho }$, the larger the correlation coefficient.

According to Chebyshev inequality, the upper bound of generalization error can be derived:

(11) $$P_{X,Y}(mr(X,Y)<0)\leq \frac{\bar{\rho }(1-S^{2})}{S^{2}}$$

It can be seen that the bound of generalization error of random forest is negatively correlated with the classification intensity *S* of a single decision tree and positively correlated with the correlation *P* between decision trees. That is, the larger the classification intensity *S*, the smaller the correlation *P*. The smaller the bound of generalization error is, the higher the classification accuracy of the random forest will be.

## 4. Intrusion Detection Technology Combined with SMOTE and Random Forest

**Figure 3:**
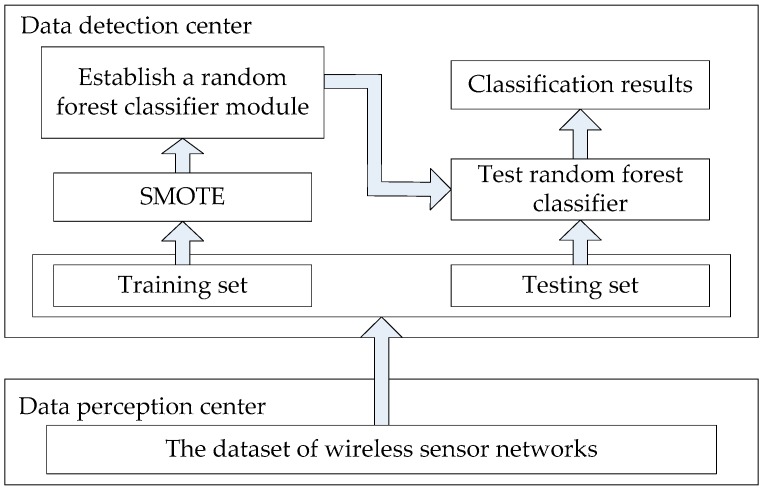
The architecture of intrusion detection system.

The intrusion detection for wireless sensor networks can be regarded as a classification problem, and the dataset can be divided into normal data and attack data. To solve the problem of class imbalance between normal data and attack data and improve the classification accuracy, SMOTE is used to oversample the dataset. After oversampling, the training set is reconstructed and the original data of class imbalance is balanced. Then the random forest algorithm is used to train the new training set, which has been balanced. Finally, the classifier is generated to realize the intrusion detection for wireless sensor networks. The architecture of intrusion detection system proposed in this paper is shown in Figure 3.

The steps of intrusion detection for wireless sensor networks based on SMOTE and random forest algorithm are as follows:

1. Suppose that the sample space of attack data of wireless sensor networks is *P* and the sample space of normal data is *Q*. *P* consists of *n* samples of attack data, and $Y_{i}$ represents the features of the ith attack data. Thus, *P* can be represented as $P=\{Y_{1},Y_{2},\cdots ,Y_{n}\}$. For each sample, there are *f* features, recorded as $Y_{i}=\{F_{i1},F_{i2},\cdots ,F_{if}\}$.
2. For each sample $Y_{i}$ in the attack data set, the Euclidean distance is used to calculate the distance from it to all other samples in *P*, and its *K* nearest neighbors are obtained.
3. The sampling magnification *N* is set according to the ratio of the number of attack data samples *P* to the number of normal data samples *Q*. *N* neighbors are randomly selected from the *K* nearest neighbors of each attack data sample $Y_{i}$, recorded as $Y_{j}^{\prime }$, where j=1,2,⋯,N.
4. Each randomly-selected neighbor sample B constructs a new attack data sample with attack data sample D according to Equation (12). The rand(0,1) represents a random number of the interval [0,1]:
  (12) $$Y_{new}=Y_{i}+rand(0,1)\cdot (Y_{j}^{\prime }-Y_{j})$$
5. Combine the constructed new samples with the normal data samples *Q* to generate a new data sample space *R*.
6. Assuming $X_{i}$ represents the ith data sample, then $R=\{X_{1},X_{2},\cdots ,X_{n}\}$. For each sample, there are *f* features, which are recorded as $X_{i}=\{F_{i1},F_{i2},\cdots ,F_{if}\}$. Select the decision tree and use it as the base classifier.
7. A new training set $R_{j}$ is generated by sampling from the data sample space *R* using the method of Bootstrap, and a decision tree is constructed by $R_{j}$.
8. The *k*(k≤f) features are randomly extracted from the nodes of each decision tree. By calculating the amount of information contained in each feature, a feature with the most classification ability is selected among the *k* features to split the nodes until the tree grows to the maximum.
9. Repeat steps 7 and 8 for *m* times to train *m* decision trees.
10. The generated decision trees are composed of random forest, and the new data is classified by the random forest. The classification results are determined by the number of votes of the tree classifiers.

The method of SMOTE + random forest takes attack data as a minority class and generates new attack data through SMOTE, which reduces the difference in the number of attack data and normal data, and reduces the imbalance of the training set. The method can obtain better classification effect and effectively improve the accuracy of intrusion detection for wireless sensor networks.

## 5. Experimental Results and Analysis

### 5.1. Dataset and Evaluation Indicators

The KDD Cup 99 dataset [44], which is widely recognized in the field of intrusion detection, is used as training and testing set. The dataset is a network traffic data set created by MIT Lincoln Laboratory by simulating the local area network environment of the U.S. Air Force. There are different probability distributions for testing data and training data, and the testing set contains some types of attacks that do not appear in the training set, which makes the intrusion detection more realistic.

**Figure 4:**
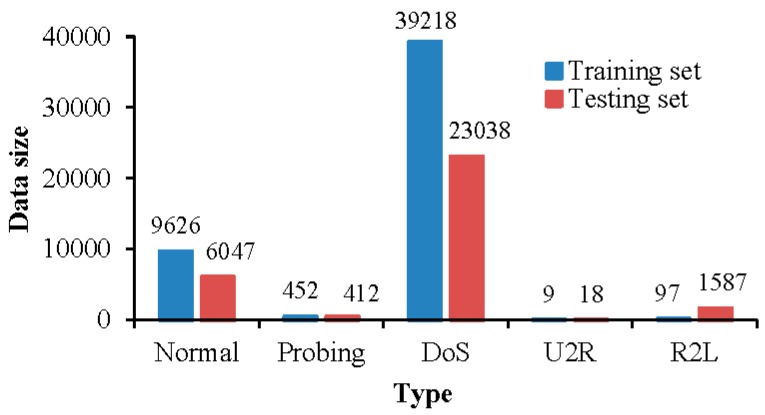
Distribution of various types of data in the dataset.

The dataset has 41 different attributes, and it can be divided into five different types, one normal type and four attack types (DoS, Probing, U2R, and R2L). Denial of service (DoS) attacks prevent legitimate requests for network resources by consuming bandwidth or overloading computational resources. Probing attack refers to when an attacker scans the network to collect information about the target system before launching an attack. User to root (U2R) attack refers to that legitimate users obtain the root access right of the system by illegal means. Root to local (R2L) attack refers to the attack method of gaining access to the local host by sending customized network packets. Since the dataset is too large, 49,402 records are randomly selected from the “10% KDD Cup 99 training set” as training data, and 31,102 records are randomly selected from the “KDD Cup 99 corrected labeled test dataset” as testing data. The distribution of various types of data in training set and testing set is shown in Figure 4.

In intrusion detection systems, accuracy, precision, AUC, etc. are usually used as indicators to evaluate the system [45,46]. Accuracy is the proportion of the records correctly classified, which is defined as follows:

(13) $$accuracy=\frac{TP+TN}{TP+TN+FN+FP}$$

Among them, *TP* refers to the number of records that attack behavior is recognized as attack behavior, *TN* refers to the number of records that normal behavior is recognized as normal behavior, *FP* refers to the number of records that normal behavior is recognized as attack behavior, *FN* refers to the number of records that attack behavior is recognized as normal behavior.

Precision is the proportion of the records that are actually attacks in the records that are predicted to attacks. Precision is higher, indicating that the false positive rate (FPR) of the system is lower. Precision is defined as follows:

(14) $$precision=\frac{TP}{TP+FP}$$

Area under the curve (AUC) is defined as the area under the ROC curve. Obviously, the value of this area will not be greater than 1. Because ROC curve is generally above the line y=x, AUC ranges from 0.5 to 1. The AUC value is used as the evaluation criterion because ROC curve cannot clearly explain which classifier is better in many cases, and AUC as a numerical value can intuitively explain that the classifier with larger AUC has better effect.

### 5.2. Results and Comparison

The experimental environment of this experiment is mainly based on Weka [47], a famous open source software for machine learning and data mining. All comparison algorithms are also derived from the data packages provided by Weka. The experiment was implemented on 2.6 GHz Intel core i5-3320M processor with 4GB RAM. In this paper, the classical single classifiers and ensemble classifiers in Weka are selected and compared. The single classifiers include J48 [48], NaiveBayes [49], LibSVM [50], and the ensemble classifiers include Bagging [51], AdaBoostM1 [52], and RandomForest [53].

The precision of each classifier is shown in Table 1. It can be seen from the table that the classification results of minority classes, such as probing, U2R, and R2L, are poor, and the problem of class imbalance is obvious. The AUC value of each classifier is shown in Table 2. It can be seen from the table that classifiers such as J48, AdaboostM1 and RandomForest have better classification effect than LibSVM, NaiveBayes and Bagging for the problem of class imbalance.

**Figure 5:**
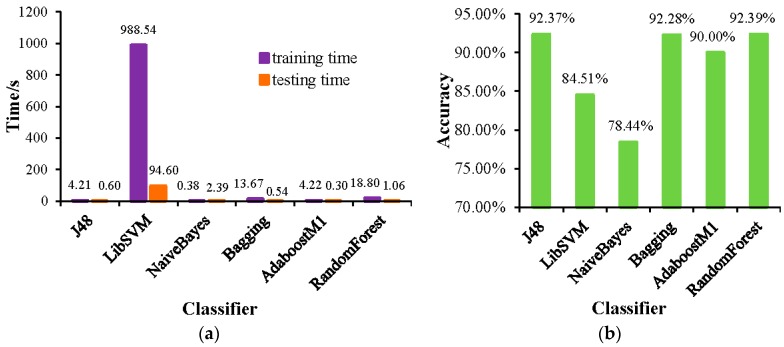
(**a**) The training time and testing time of each classifier; and (**b**) the accuracy of each classifier.

The training time and testing time of each classifier are shown in Figure 5a. It can be seen from the figure that the training and testing time of LibSVM classifier is much longer than other classifiers, and the data processing speed is slower. The accuracy of each classifier is shown in Figure 5b. It can be seen from the figure that the accuracy of the J48, Bagging, and RandomForest classifiers is high, especially the classification effect of the RandomForest classifier is the best.

Since the classification effect of minority classes, such as probing, U2R, and R2L, is poor, the method of SMOTE is used to solve the problem of class imbalance. In order to verify the effect of the previous six classifiers combined with the SMOTE, the classifiers are tested with the SMOTE respectively. The precision and AUC value of each classifier combined with the SMOTE are shown in Table 3 and Table 4, respectively. It can be seen from the table that values of precision and AUC have been improved.

**Figure 6:**
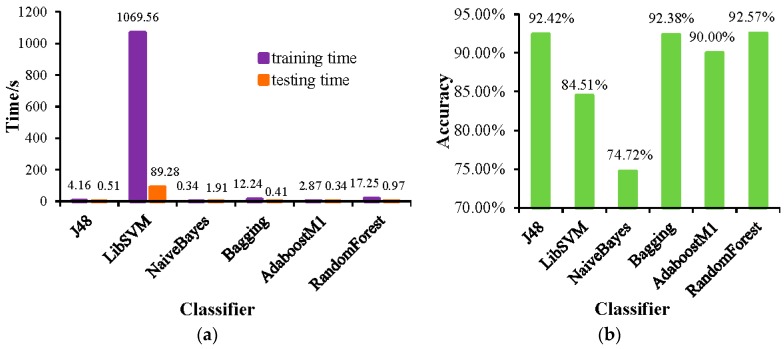
(**a**) The training time and testing time of each classifier combined with the SMOTE; and (**b**) the accuracy of each classifier combined with SMOTE.

The training time and testing time of each classifier combined with the SMOTE are shown in Figure 6a. It can be seen from the figure that the training time and testing time of each classifier are significantly shortened. The accuracy of each classifier combined with the SMOTE is shown in Figure 6b. It can be seen from the figure that the accuracy of Bagging and RandomForest classifiers has been improved after using the method of SMOTE. In this experiment, the accuracy of the method of SMOTE+RandomForest reaches 92.57%, which is the best performance of all methods.

Due to the similar accuracy between J48 and RandomForest, the comparative experiments under datasets with different sampling proportions are carried out. Five different datasets are randomly selected from “10% KDD Cup 99 training set” and “KDD Cup 99 corrected labeled test dataset” according to 5%, 7.5%, 10%, 12.5%, and 15% sampling proportions. The amount of training data and testing data in each dataset is shown in Table 5.

**Figure 7:**
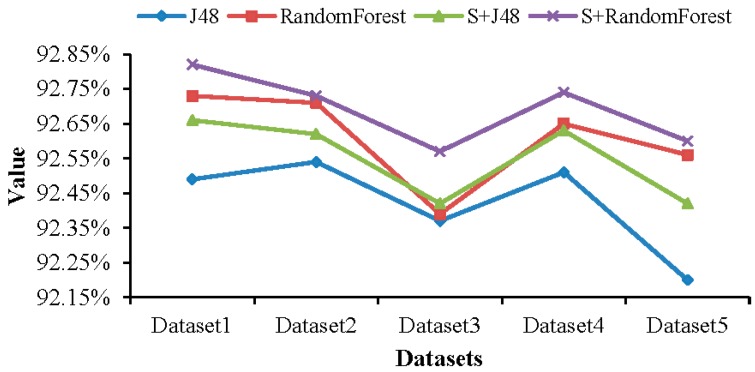
Comparison of the performance of J48, RandomForest, S+J48, and S+RandomForest under different proportions of datasets.

The comparison of the performance of J48, RandomForest, S+J48, and S+RandomForest under different proportions of datasets is shown in Figure 7. It can be seen from the figure that the accuracy of RandomForest is better than that of J48, the accuracy of J48 and RandomForest combined with SMOTE is higher than that without SMOTE.

## 6. Conclusions

The intrusion detection for wireless sensor networks is an important subject in the field of the security of wireless sensor networks. Due to the class imbalance in KDD Cup 99 dataset, this study combines the SMOTE with the random forest algorithm, and proposes an ensemble classifier for imbalanced datasets. Experiments on KDD Cup 99 dataset show that the classification accuracy of random forest algorithm has reached 92.39%, which is higher than other classification methods, such as J48, LibSVM, NaiveBayes, Bagging, and AdaboostM1. After combining with the SMOTE, the classification accuracy of the random forest has increased to 92.57%, which improves the classification effect of minority classes. The random forest method combined with the SMOTE provides an effective solution to solve the problem of class imbalance and improves the classification accuracy of intrusion detection. Moreover, this method is simple to implement and has strong generalization ability. It can be widely used in the field of the security of wireless sensor networks to improve the effect of intrusion detection for wireless sensor networks. In the future, this research will continue to find new classification methods to further improve the recognition effect of the intrusion data of wireless sensor networks.

## Figures and Tables

**Table 1:** The precision of each classifier.

Type\Classifier	J48	LibSVM	NaiveBayes	Bagging	AdaboostM1	RandomForest
Normal	0.728	0.562	0.730	0.758	0.682	0.728
Probing	0.808	0.697	0.083	0.890	0.000	0.896
DoS	0.998	0.997	0.992	0.982	0.984	0.995
U2R	0.000	0.000	0.049	0.000	0.000	1.000
R2L	0.983	0.000	0.771	0.680	0.000	0.990

**Table 2:** The AUC value of each classifier.

Type\Classifier	J48	LibSVM	NaiveBayes	Bagging	AdaboostM1	RandomForest
Normal	0.951	0.904	0.970	0.979	0.950	0.974
Probing	0.890	0.686	0.978	0.924	0.919	0.993
DoS	0.980	0.933	0.896	0.995	0.971	0.990
U2R	0.644	0.500	0.519	0.908	0.928	0.935
R2L	0.804	0.500	0.949	0.567	0.888	0.669

**Table 3:** The precision of each classifier combined with the SMOTE.

Type\Classifier	J48	LibSVM	NaiveBayes	Bagging	AdaboostM1	RandomForest
Normal	0.725	0.562	0.809	0.759	0.682	0.728
Probing	0.904	0.697	0.086	0.453	0.000	0.901
DoS	0.998	0.997	0.886	0.994	0.984	0.999
U2R	0.375	0.000	0.044	0.200	0.000	0.333
R2L	0.941	0.000	0.717	0.944	0.000	0.981

**Table 4:** The AUC value of each classifier combined with the SMOTE.

Type\Classifier	J48	LibSVM	NaiveBayes	Bagging	AdaboostM1	RandomForest
Normal	0.949	0.904	0.970	0.974	0.943	0.976
Probing	0.891	0.686	0.981	0.868	0.774	0.995
DoS	0.982	0.933	0.892	0.987	0.970	0.986
U2R	0.720	0.500	0.542	0.856	0.869	0.995
R2L	0.519	0.500	0.949	0.571	0.921	0.677

**Table 5:** The amount of training data and testing data in each dataset.

Type\Dataset	Dataset1	Dataset2	Dataset3	Dataset4	Dataset5
Training data	24,701	37,051	49,402	61,752	74,103
testing data	15,551	23,327	31,102	38,878	46,654

## References

[1] Zhang Z., Glaser S., Bales R., Conklin M., Rice R., Marks D. (2017). Technical report: The design and evaluation of a basin-scale wireless sensor network for mountain hydrology. Water Resour. Res..

[2] Victor G.F., Carles G., Helena R.P. (2016). A Comparative Study of Anomaly Detection Techniques for Smart City Wireless Sensor Networks. Sensors.

[3] Lu T., Liu G., Chang S. (2018). Energy-efficient data sensing and routing in unreliable energy-harvesting wireless sensor network. Wirel. Netw..

[4] Fang X., Nan L., Jiang Z., Chen L. (2017). Fingerprint localisation algorithm for noisy wireless sensor network based on multi-objective evolutionary model. IET Commun..

[5] Wang J., Jiang S., Fapojuwo A.O. (2017). A Protocol Layer Trust-Based Intrusion Detection Scheme for Wireless Sensor Networks. Sensors.

[6] Ferng H.W., Khoa N.M. (2017). On security of wireless sensor networks: A data authentication protocol using digital signature. Wirel. Netw..

[7] Ismail B., In-Ho R., Ravi S. (2015). An Intrusion Detection System Based on Multi-Level Clustering for Hierarchical Wireless Sensor Networks. Sensors.

[8] Li M., Lou W., Ren K. (2016). Data security and privacy in wireless body area networks. IEEE Wirel. Commun..

[9] Ren J., Zhang Y., Zhang K., Shen X. (2016). Adaptive and Channel-Aware Detection of Selective Forwarding Attacks in Wireless Sensor Networks. IEEE Trans. Wirel. Commun..

[10] Wang J., Wang F., Cao Z., Lin F., Wu J. (2017). Sink location privacy protection under direction attack in wireless sensor networks. Wirel. Netw..

[11] Xiao X., Zhang R. (2017). Study of Immune-Based Intrusion Detection Technology in Wireless Sensor Networks. Arab. J. Sci. Eng..

[12] Yan J., Li X., Luo X., Guan X. (2017). Virtual-Lattice Based Intrusion Detection Algorithm over Actuator-Assisted Underwater Wireless Sensor Networks. Sensors.

[13] Kalnoor G., Agarkhed J. (2018). Detection of Intruder using KMP Pattern Matching Technique in Wireless Sensor Networks. Proc. Comput. Sci..

[14] Osanaiye O.A., Alfa A.S., Hancke G.P. (2018). Denial of Service Defence for Resource Availability in Wireless Sensor Networks. IEEE Access.

[15] Ma T., Wang F., Cheng J., Yu Y., Chen X. (2016). A Hybrid Spectral Clustering and Deep Neural Network Ensemble Algorithm for Intrusion Detection in Sensor Networks. Sensors.

[16] Wazid M., Das A. (2016). An Efficient Hybrid Anomaly Detection Scheme Using K-Means Clustering for Wireless Sensor Networks. Wirel. Pers. Commun..

[17] Belavagi M.C., Muniyal B. (2016). Performance Evaluation of Supervised Machine Learning Algorithms for Intrusion Detection. Proc. Comput. Sci..

[18] Lu N., Sun Y., Liu H., Li S. (2018). Intrusion Detection System Based on Evolving Rules for Wireless Sensor Networks. J. Sens..

[19] Singh R., Singh J., Singh R. (2017). Fuzzy Based Advanced Hybrid Intrusion Detection System to Detect Malicious Nodes in Wireless Sensor Networks. Wirel. Commun. Mob. Comput..

[20] Sun Z., Xu Y., Liang G., Zhou Z. (2018). An Intrusion Detection Model for Wireless Sensor Networks with an Improved V-Detector Algorithm. IEEE Sens. J..

[21] Tajbakhsh A., Rahmati M., Mirzaei A. (2009). Intrusion detection using fuzzy association rules. Appl. Soft. Comput..

[22] Xie M., Hu J., Guo S., Zomaya A.Y. (2017). Distributed Segment-Based Anomaly Detection with Kullback–Leibler Divergence in Wireless Sensor Networks. IEEE Trans. Inf. Forensic Secur..

[23] Xie M., Hu J., Guo S. (2015). Segment-based anomaly detection with approximated sample covariance matrix in wireless sensor networks. IEEE Trans. Parallel Distrib. Syst..

[24] Haider W., Hu J., Slay J., Turnbull B.P., Xie Y. (2017). Generating realistic intrusion detection system dataset based on fuzzy qualitative modeling. J. Netw. Comput. Appl..

[25] Ye Y., Li T., Adjeroh D., Iyengar S.S. (2017). A survey on malware detection using data mining techniques. ACM Comput. Surv..

[26] Kumar M., Hanumanthappa M. (2013). Intrusion detection system using stream data mining and drift detection method. Res. Vet. Sci..

[27] Khorshidpour Z., Hashemi S., Hamzeh A. (2017). Evaluation of random forest classifier in security domain. Appl. Intell..

[28] Paul A., Mukherjee D.P., Das P., Gangopadhyay A., Chintha A.R., Kundu S. (2018). Improved Random Forest for Classification. IEEE Trans. Image Process..

[29] Lee S.M., Kim D.S., Park J.S. (2011). A Hybrid Approach for Real-Time Network Intrusion Detection Systems. IEEE Trans. Veh. Technol..

[30] Singh K., Guntuku S.C., Thakur A., Hota C. (2014). Big Data Analytics framework for Peer-to-Peer Botnet detection using Random Forests. Inf. Sci..

[31] Ronao C.A., Cho S.B. (2016). Anomalous query access detection in RBAC-administered databases with random forest and PCA. Inf. Sci..

[32] Taft L.M., Evans R.S., Shyu C.R., Egger M.J., Chawla N., Mitchell J.A., Thornton S.N., Bray B., Varner M. (2009). Countering imbalanced datasets to improve adverse drug event predictive models in labor and delivery. J. Biomed. Inform..

[33] Sun J., Shang Z., Li H. (2014). Imbalance-oriented SVM methods for financial distress prediction: A comparative study among the new SB-SVM-ensemble method and traditional methods. J. Oper. Res. Soc..

[34] Santos M.S., Abreu P.H., García-Laencina P.J., Simão A., Carvalho A. (2015). A new cluster-based oversampling method for improving survival prediction of hepatocellular carcinoma patients. J. Biomed. Inform..

[35] Chawla N.V., Bowyer K.W., Hall L.O., Kegelmeyer W.P. (2002). SMOTE: Synthetic minority over-sampling technique. J. Artif. Intell. Res..

[36] Lusa L. (2013). SMOTE for high-dimensional class-imbalanced data. BMC Bioinform..

[37] Jeatrakul P., Wong K.W., Fung C.C. Classification of imbalanced data by combining the complementary neural network and SMOTE algorithm. Proceedings of the International Conference on Neural Information Processing.

[38] Wang J., Xu M., Wang H., Zhang J. Classification of imbalanced data by using the SMOTE algorithm and locally linear embedding. Proceedings of the International Conference on Signal Processing.

[39] Blagus R., Lusa L. Evaluation of smote for high-dimensional class-imbalanced microarray data. Proceedings of the International Conference on Machine Learning and Applications.

[40] Breiman L. (1996). Bagging predictors. Mach. Learn..

[41] Hasan M.A., Nasser M., Ahmad S., Molla M.K. (2016). Feature Selection for Intrusion Detection Using Random Forest. J. Inf. Secur..

[42] Farnaaz N., Jabbar M.A. (2016). Random forest modeling for network intrusion detection system. Proc. Comput. Sci..

[43] Yi Y.A., Min M.M. An analysis of random forest algorithm based network intrusion detection system. Proceedings of the International Conference on Software Engineering, Artificial Intelligence, Networking and Parallel/Distributed Computing.

[44] KDD Cup 1999 Data. http://kdd.ics.uci.edu/databases/kddcup99/kddcup99.html.

[45] Liu Y., Xiang C., Wang H. (2017). Optimization of feature selection based on mutual information in intrusion detection. J. Northwest. Univ..

[46] Yan J.H. (2018). Optimization Boosting Classification Based on Metrics of Imbalanced Data. Comput. Eng. Appl..

[47] Hall M., Frank E., Holmes G., Pfahringer B., Reutemann P., Witten I.H. (2009). The WEKA data mining software: An update. ACM SIGKDD Explor. Newsl..

[48] Sahu S., Mehtre B.M. Network intrusion detection system using J48 Decision Tree. Proceedings of the International Conference on Advances in Computing, Communications and Informatics.

[49] Amor N.B., Benferhat S., Elouedi Z. Naive Bayes vs decision trees in intrusion detection systems. Proceedings of the ACM Symposium on Applied Computing.

[50] Chang C.C., Lin C.J. (2011). LIBSVM: A library for support vector machines. ACM Trans. Intell. Syst. Technol..

[51] Gaikwad D.P., Thool R.C. Intrusion Detection System Using Bagging Ensemble Method of Machine Learning. Proceedings of the International Conference on Computing Communication Control & Automation.

[52] Cortes E.A., Martinez M.G., Rubio N.G. (2007). Multiclass corporate failure prediction by Adaboost.M1. Int. Adv. Econ. Res..

[53] Breiman L. (2001). Random forests. Mach. Learn..

